# The Relationship between *TLR3* rs3775291 Polymorphism and Infectious Diseases: A Meta-Analysis of Case-Control Studies

**DOI:** 10.3390/genes14071311

**Published:** 2023-06-21

**Authors:** Marcos Jessé Abrahão Silva, Caroliny Soares Silva, Marcelo Cleyton da Silva Vieira, Pabllo Antonny Silva dos Santos, Cristiane Cunha Frota, Karla Valéria Batista Lima, Luana Nepomuceno Gondim Costa Lima

**Affiliations:** 1 Graduate Program in Epidemiology and Health Surveillance (PPGEVS), Evandro Chagas Institute (IEC), Ananindeua 67030-000, PA, Brazil; jesseabrahao10@gmail.com; 2 Postgraduate Program in Parasitic Biology in the Amazon (PPGBPA), University of State of Pará (UEPA), Belém 66087-670, PA, Brazil; karolinysoares2303@gmail.com (C.S.S.); marcelocleiton14@hotmail.com (M.C.d.S.V.); antonnypabllo@gmail.com (P.A.S.d.S.); 3 Department of Pathology and Legal Medicine, Faculty of Medicine, Federal University of Ceará (UFC), Fortaleza 60441-750, CE, Brazil; cristianefrota71@gmail.com; 4 Bacteriology and Mycology Section of the Evandro Chagas Institute (IEC), Ananindeua 67030-000, PA, Brazil; karlalima@iec.gov.br

**Keywords:** *TLR3*, single nucleotide polymorphism, infectious diseases

## Abstract

As the host’s first line of defense against pathogens, *Toll-like receptors* (*TLRs*), such as the *TLR3*, are genes encoding transmembrane receptors of the same name. Depending on their expression, TLRs cause a pro- or anti-inflammatory response. The purpose of the article was to determine whether there is an association between the *Toll-like receptor 3* (*TLR3*) rs3775291 Single Nucleotide Polymorphism—SNP and susceptibility to infections. This review was conducted according to the Preferred Reporting Items for Systematic Reviews and Meta-Analyses (PRISMA) 2020 guidelines and was registered in PROSPERO under the code CRD42023429533. A systematic search for relevant studies was performed using PubMed, Scopus, SciELO, Google Scholar, and Science Direct by the MeSH descriptors and the Boolean Operator “AND”: “Infections”; “TLR3”; “SNP”, between January 2005 and July 2022. Summary odds ratios (ORs) and corresponding 95% confidence intervals (CIs) were calculated for genotypic comparison assuming a dominant genetic model (CT + TT vs. CC). A meta-analysis of 18 studies consisting of 3118 cases and 4368 controls found a significant association for risk between the presence of the *TLR3* SNP rs3775291 and infections as part of the general analysis (OR = 1.16, 95% CI = 1.04–1.28, *p* = 0.004). In the subgroups of continents, the SNP had a protective role in Europe for 1044 cases and 1471 controls (OR = 0.83, 95% CI = 0.70–0.99, *p* = 0.04); however, the Asian (for 1588 patients and 2306 controls) and American (for 486 patients and 591 controls) continents had an increase in infectious risk (OR = 1.37, 95% CI = 1.19–1.58, *p* < 0.001; OR = 1.42, 95% CI = 1.08–1.86, and *p* = 0.01, respectively). Heterogeneity between studies was detected (I^2^ = 58%) but was explained in meta-regression by the subgroup of continents itself and publication bias was not evident. The results of the meta-analysis suggest a significant association between the *TLR3* rs3775291 polymorphism and susceptibility to infections. Thus, when analyzing subgroups, the Asian and American continents showed that this SNP confers a higher risk against infections in a dominant genotypic model. Therefore, more studies are necessary to fully elucidate the role of *TLR3* rs3775291 in infections.

## 1. Introduction

Since they are closely related to deprivation and unhealthy living conditions, infectious diseases pose a serious public health concern. In this sense, a pattern can be seen in the epidemiological indices of various diseases in relation to the development of the population and the quality of life in a particular area [1]. In the world, they are the second leading cause of death [2].

There are certain characteristics of clinical presentation, such as symptomatic and asymptomatic patients, in an endemic community for a given disease. Immunological variability, which is mainly caused by changes in genetic background, is one of the causes of this, among other factors [3]. Therefore, to fully understand the various consequences of infections, either through susceptibility or protection against them, associative immunogenetic investigations are crucial [4].

Animals’ innate immunity is dependent on pattern recognition receptors (PRRs), which are specialized in recognizing pathogen-associated molecular patterns (PAMPs) and then activating a signaling pathway to cause type I interferon (IFN-I)- and interleukin-1 (IL-1)-mediated pro-inflammatory reactions [5].

*Toll-like receptors* (*TLRs*) are transmembrane receptor genes that are PRRs that are found in endosomes or on the surfaces of immune cells [6]. These receptors mediate the production of cytokines necessary for effective immunization while also detecting pathogen-associated molecular patterns (PAMPs) and damage-associated molecular patterns (DAMPs) in cells [7]. There can be several types of signaling pathways activated based on PAMP. Each member of this receptor family (TLR1-10) has different expression patterns in various organs and particular ligands to carry out this identification in humans [8]. TLRs are located mainly on the surfaces of macrophages and dendritic cells (DCs), among other cell types, as well as on the membranes of endosomes and lysosomes [9].

A double-stranded RNA (dsRNA)-TLR3 signaling complex, consisting of one dsRNA and two TLR3 molecules, is created when TLR3 binds to dsRNA. *TLR3* is a gene that encodes the protein of the same name with 904 amino acids, which is responsible for recognizing the dsRNA of infectious agents, a viral replication intermediate, in cellular endosomes. *TLR3* begins downstream signal transmission and induces the creation of the antiviral protein (AVP). This gene is located on the human chromosome 4q35.1 and has five exons (coding regions) [10].

Numerous epithelial cells, including fibroblasts, immune cells, neurocytes, and immune cells, carry TLR3, which is most widely expressed in the placenta and pancreas. TLR3 works through the TIR domain-containing adaptor-inducing IFN-β (TRIF)-dependent TLR signaling pathway and acts on DCs bearing antigens responsible for inducing antigen-specific immune responses mediated by lymphocytes [11].

Among the most varied types of polymorphism, single nucleotide polymorphism (SNP) is a punctual change of nucleotide that can occur in introns (noncoding regions) or exons [12]. In this sense, the *TLR3* SNP rs3775291 is a non-synonymous mutation (Cytosine to Thymine, C > T) of the missense type in exon 4, that is, it causes a change in the codon of amino acids from leucine (Leu) to phenylalanine (Phe) at residue 412 and its presence results in hypoactivity of the receptor in the human organism [13].

The level of *TLR3* transcript is not affected by this SNP, but it was discovered to decrease the ability of *TLR3* to attach to dsRNA [13]. The function of *TLR3* is only partially compromised by the substitution of Leu412Phe, resulting in an attenuated inflammatory reaction. The solenoid protein structure becomes unstable if 412Phe is present, and this alters any possible glycosylation of the nearby residue Asn413 (which was found to have N-acetylglucosamines attached). In this case, the ectodomain of the *TLR3* receptor is formed by the Leu412Phe variation, which is close to the glycosylation location (Asn413) and a crucial region for the dimerization of the domain at the membrane [14].

Investigating *TLR3* SNPs and diseases for the characterization of biomarkers in populations is of enormous immunogenetic relevance in this context. Using case-control epidemiological research, this work seeks to summarize and assess the relationship between the *TLR3* SNP rs3775291 and infectious illnesses. Because bias can occur in original studies as a result of flaws in the design of the included study, which tend to skew the magnitude or direction of associations in the data, the case-control study design was chosen as the selection strategy for the research added to this meta-analysis [15].

## 2. Material and Methods

### 2.1. Study Design

This systematic review was conducted in accordance with the Preferred Reporting Items for Systematic Reviews and Meta-Analyses (PRISMA) 2020 statement [16]. To create the guiding question, the PICO strategy was used with the following anagrams: population, intervention, comparison, and outcome. In this context, it was developed from Population: patients with infectious diseases; Intervention: association between *TLR3* SNP rs3775291 and infectious diseases; Comparison: infectious diseases and *TLR3* SNP rs3775291; Outcome: identification of susceptibility or protective functions of *TLR3* SNP rs3775291 for infectious diseases published in the literature [17]. This review was registered in PROSPERO under the code CRD42023429533.

### 2.2. Search Strategy

The identification and selection of articles were performed in the databases Science Direct, the National Library of Medicine National Institutes of Health of the USA (PUBMED), Google Scholar, Scientific Electronic Library Online (SciELO), and Scopus using the descriptors: “*TLR3*”, “Infections”, and “Single Nucleotide Polymorphism”, together with the Boolean operator “AND”. The time cutoff was designated from the beginning of publications on the *TLR3* SNP rs3775291 (January 2005) until July 2022. The data were collected on 21 July 2022. The languages included in the study were limited to English, Portuguese, and Spanish.

The study titles and abstracts were examined and those that included *TLR3* polymorphisms and the probability of contracting an infectious disease were considered for a more thorough analysis. Electronic surveys were conducted from inception to 21 July 2020. Studies that examined *TLR3* polymorphisms and their link to noninfectious illness, as well as studies that were not published in English, were also eliminated. A study was considered eligible if it met all three of the following requirements: (i) it revealed an infectious disease outcome; (ii) it was carried out using a case-control design, where “cases” are people who have the disease outcome and “controls” are people in the healthy population who do not; (iii) it revealed genotype frequencies for *TLR3* rs3775291 (+1234C/T); (iv) the genotyping method by molecular biology.

### 2.3. Data Extraction

Two authors (MJAS and CSS) independently extracted all data that were considered relevant, including the differences and ambiguities found in the publications, and in cases of inconsistent selection, a third author (MCSV) participated in data selection. The information extracted included the name of the author, the origin of the population, the number of cases and controls subdivided by genotype frequencies (wild-type, heterozygous, and mutant homozygous), the disease being studied, and the conclusions reportedly drawn from each study.

Data extraction was conducted concurrently with an evaluation of the study’s quality and bias by the ROBINS-E risk tool (Risk of Bias In Non-randomized Studies—of Exposures). Review Manager version 5.4 (Nordic Cochrane Centre, Cochrane Collaboration, Copenhagen, Sweden) was used to perform this analysis. Any disagreement between the two analyses of the researchers was discussed by a third author (MCSV) with regard to the inclusion of the study, any uncertainties in the data extraction process, and the quality and risk of bias evaluation within the larger group (MJAS and CSS).

### 2.4. Statistical Analysis

The Comprehensive Meta-Analyses—CMA program, version 2.2 (Biostat, Englewood, NJ, USA) was used on computer to perform the statistical analysis of the meta-analysis for the investigation. The fixed effects model estimated summary odds ratios (ORs) with 95% confidence intervals (95% CI). In each study’s controls, the Hardy–Weinberg equilibrium (HWE) was evaluated using the chi-square (Q) goodness-of-fit test. Only genotypic comparison was performed using a dominant genetic model (genotypes CT and TT vs. CC). Using the I^2^ statistics, the heterogeneity between studies was evaluated for comparisons.

According to Cochrane, the level of heterogeneity in a meta-analysis is analyzed based on the following parameters: 0% to 40%, it may not be important; 30% to 60% may represent moderate heterogeneity; 50% to 90% may represent substantial heterogeneity [18]. The chi-square test is one of the most commonly used tests to assess the significance of heterogeneity, with a significance level of *p* < 0.05 being used [19]. A subgroup analysis was performed according to the Continent. The Cochrane Q-test and I-squared (I^2^) measure were used to determine the statistical difference groups (*p* < 0.05 was considered statistically significant). Begg’s rank correlation test and a funnel plot were used to examine the potential for publication bias (*p* < 0.05 was considered statistically significant). Sensitivity analysis, meta-regression, and subgroup analysis based on study location were used to assess potential causes of variability, where applicable.

## 3. Results

### 3.1. Literature Search

**Figure 1:**
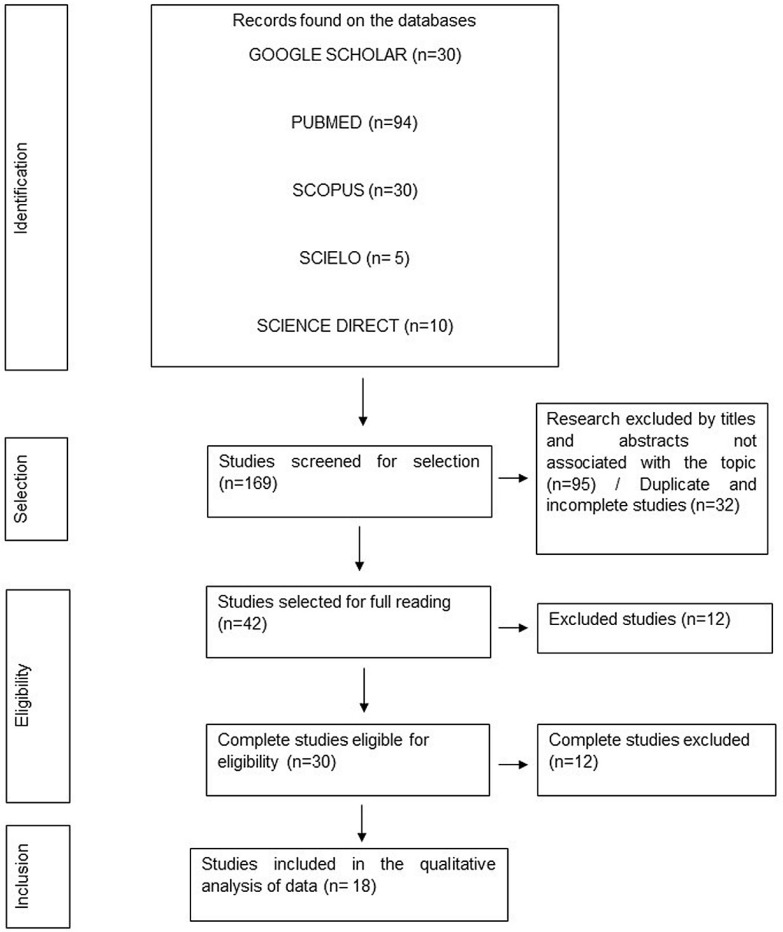
PRISMA flowchart representing the stages of selection, eligibility, and inclusion of studies for analysis. Belém, PA, Brazil (2022).

Figure 1 summarizes the selection process. The search in four databases identified 169 articles, with only 32 nonduplicated or incomplete works. Subsequently, 95 articles were excluded based on titles and abstracts not relevant to the topic’s theme or not associated with the searched SNP. Therefore, 42 articles were selected for complete reading, of which 12 were removed due to the type of study that did not correspond to the case-control. Regarding this number, 30 studies were eligible for inclusion; however, 12 were withdrawn due to incomplete data on the frequency of SNP allele or genotype in study subjects. Thus, this meta-analysis consisted of 18 case-control studies, mainly from PUBMED, and studies found in all languages covered by the methodology were included.

**Figure 2:**
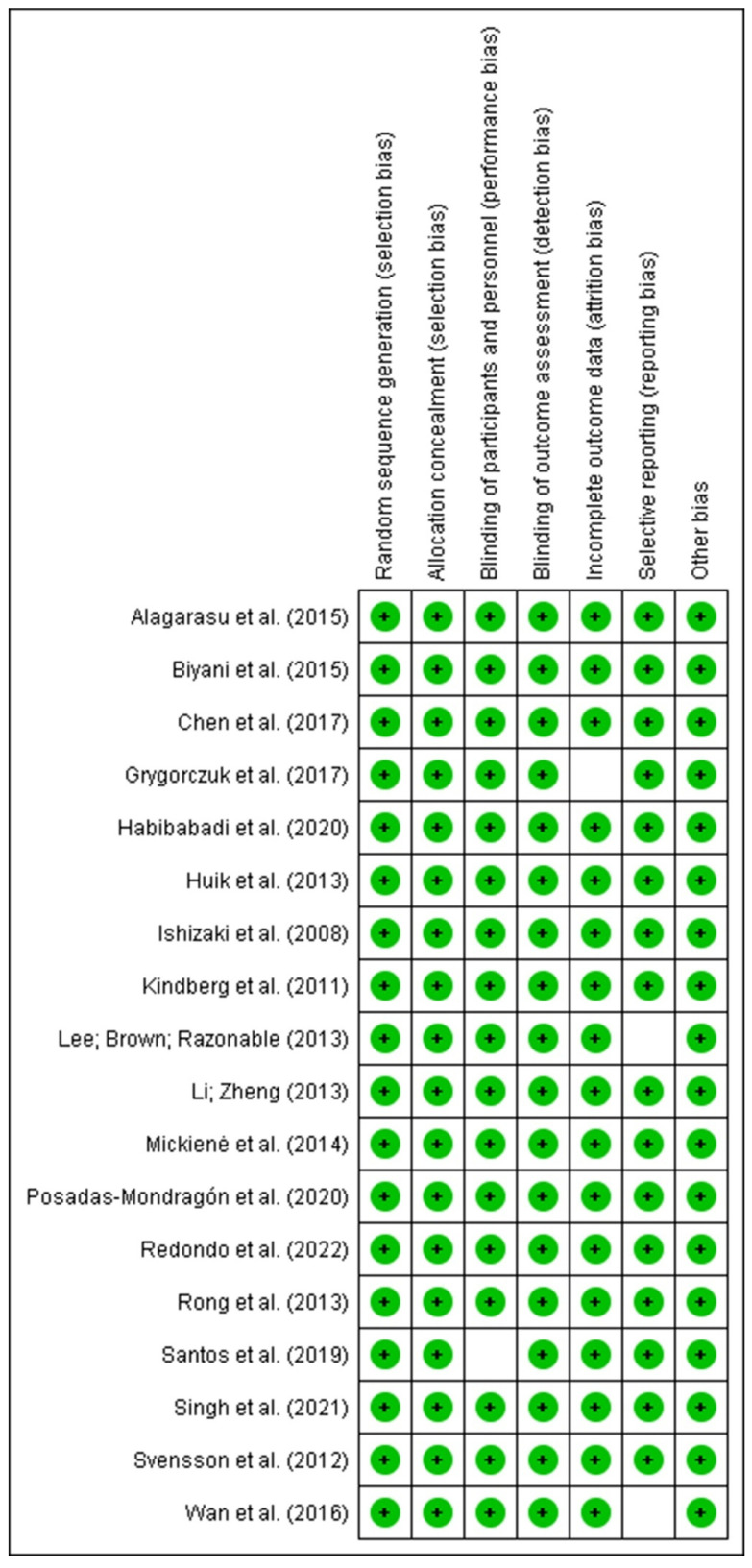
Risk of bias summary: review authors’ judgments about each risk of bias item for each included study. Symbols in green mean compliance with the prerogative of that attribute investigated for that study, while the blank spaces (empty) demonstrate the gap for that information, and those in red indicate high methodological disagreement [13,20,21,22,23,24,25,26,27,28,29,30,31,32,33,34,35,36]. Source: Elaborated by the authors with RevMan v5.4 software.

Figure 2 shows the composition of the articles included in terms of methodological quality assessment of the risk of bias in each. The evaluation was carried out separately by two evaluators in accordance with the uniform quality standard and was then cross-checked. When they ran into disagreements, they talked things out or asked the third author for guidance.

### 3.2. Characteristics of the Included Studies

The basic characteristics of the studies, including the relationship between the *TLR3* rs3775291 polymorphism and the risk of infections, are found in Table 1. The data extracted came from surveys with populations from 12 countries on 3 continents (European, Asian, and American). The origin of the studies was in descending order, Asia (nine studies), Europe (six studies), and America (three studies). For the continent, most of the added studies were Asian (equivalent to 33.33%), while for the analysis towards countries, Chinese was the most added (*n* = 4, 22.22%) (Table 1).

### 3.3. Results of a Meta-Analysis and Publication Bias between the TLR3 rs3775291 Polymorphism and the Risk of Infections in Subgroups of Continents

**Figure 3:**
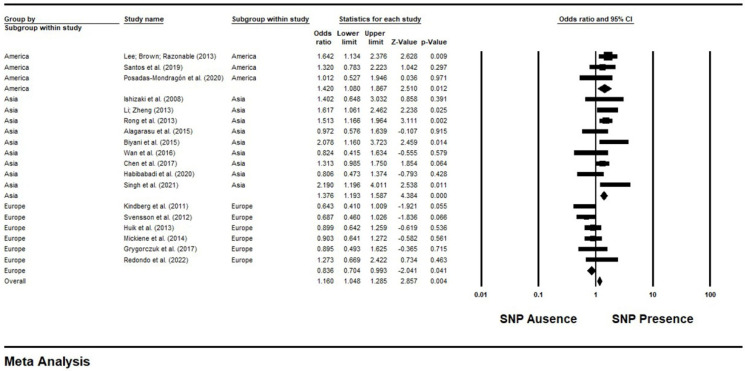
Forest plot of comparison about *TLR3* SNP rs3775291 and risk of infections, outcome: SNP presence for genotypes CT/TT vs. CC. The OR of each study is represented on the plot as a square with the area of each square proportional to the weight of the corresponding study in the meta−analysis. Horizontal lines are the 95% CIs associated for the OR of each study. The bold values highlight the total frequency of cases and controls, as well as the overall OR and the 95% CI [13,20,21,22,23,24,25,26,27,28,29,30,31,32,33,34,35,36]. Source: Elaborated by the authors with Comprehensive Meta−Analyses v2.2 software.

Meta-analysis was performed using a fixed effect, and the overall effect estimate is plotted as a diamond. In a general analysis, a significant correlation was found directed toward the higher risk of infections in 3118 cases and 4368 controls (OR = 1.16, 95% CI = 1.04–1.28, *p* = 0.004). The subgroup analysis of the six studies conducted in European populations (*n* = 1044 cases and 1471 controls) and the general estimates indicate the association between protection from infections and the presence of the mutant allele of this SNP (OR = 0.83, 95% CI = 0.70–0.99, *p* = 0.04). Genotypic comparisons for the analyses of Asian (for 1588 patients and 2306 controls) and American (for 486 patients and 591 controls) subgroups were both statistically significant for the higher risk of these diseases investigated in the literature (OR = 1.37, 95% CI = 1.19–1.58, *p* < 0.001; OR = 1.42, 95% CI = 1.08–1.86, *p* = 0.01, respectively) (Figure 3).

**Figure 4:**
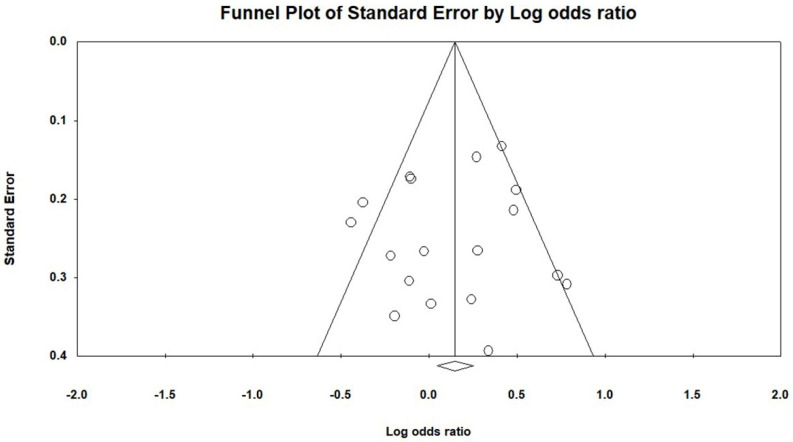
Funnel plot of comparison of the *TLR3* SNP rs3775291 and infection risk for all studies included in the meta-analysis, outcome: SNP presence for genotypes. Circles represent the included published studies and should be symmetrically dispersed around the overall effect in the form of an inverted funnel. Studies with higher precision are closer to the true value and situated at the narrowest part of the funnel. On the Y-axis of the graph, there is a measure of dispersion, the standard error, which is influenced by the sample size of the study. The larger this value is, the greater the inaccuracy of the study. On the X-axis of the graph, there is the effect measure measured in the meta-analysis and the center line is the result of this (which is directed on the X-axis by the diamond). The lines that make up the outline of the funnel correspond to the 95% CI. Source: Elaborated by the authors with Comprehensive Meta−Analyses v2.2 software.

The standard error of the logarithm of the OR (SE(log[OR])) was plotted against the OR for each study. According to a widely accepted interpretation, when selection bias is present, the plot will become asymmetrical and the meta-analysis’s overall impact will be skewed [20]. The symmetry, as in this study, in an inverted funnel shape implies the absence of publication bias (Figure 4).

### 3.4. Subgroup and Univariate Meta-Regression Analyses

**Figure 5:**
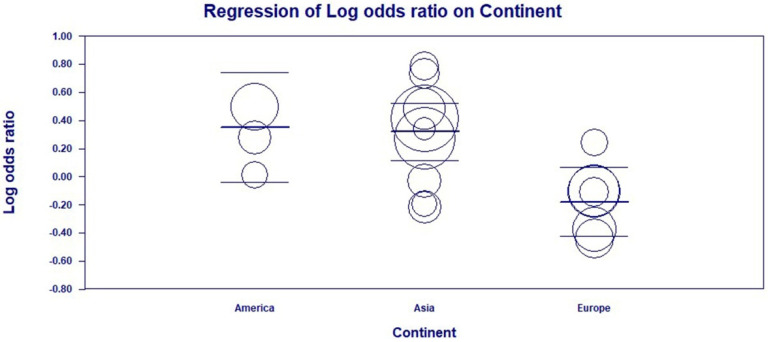
Bubble plot of the meta-regression analysis on the relationship between continent and the risk of infection based on the presence of the rs3775291 SNP. The size of the bubble is inversely related to the variance of the study. The solid line represents the linear regression (continent as the meta-independent variable). The two lines in the horizontal area between the main line correspond to the confidence intervals of the prediction. Source: Elaborated by the authors with Comprehensive Meta−Analyses v2.2 software.

Based on the generalized results found for this meta-analysis, a high level of heterogeneity was found between the included studies (Q = 40.58; *p* < 0.001; I^2^ = 58%). On the other hand, the investigation by subgroups based on continents revealed a low heterogeneity, referring to America (Q = 1.69; *p* = 0.42; I^2^ = 0%), Europe (Q = 4.29; *p* = 0.50; I^2^ = 0%), and Asia (Q = 13.07; *p* = 0.10; I^2^ = 38%). Despite this, a meta-regression analysis using a fixed model was conducted to confirm whether this factor was indeed the source of the observed heterogeneity. Figure 5 provides a visual observation of the Logit event rate by studying continent covariate (Q = 21.52; *p* < 0.001).

## 4. Discussion

TLR3 is a member of a family of immune receptors that are crucial for activating the innate immune response, indirect activation of the adaptive immune system, and control of cytokine expression in the defense of the body against infections [19,21,22]. TLR3 detects dsRNA, a molecular signature present in most viruses. TLR3 is critical for the induction of the antiviral state and the prevention of virus replication, but it can also promote an overactive and dysregulated immune response to infection, which is damaging to the host and helps to progress the severe form of the disease [23].

The presence of polymorphisms in *TLR3* is associated with changes in its structure and function, which can influence the immune response to viruses [24]. The TLR3–TRIF axis is essential in deciding how the balance between antiviral and immune regulatory pathways affects defensive versus offensive responses in the case of chronic viral infections of RNA that result in prolonged IFN-α/β signaling [25]. All articles on this *TLR3* SNP included in the meta-analysis were on viral infections. This is probably due to the binding of this receptor to its main ligand (dsRNA) in pathogens.

In this context, the molecular structure of a signaling unit (SU) reveals that dsRNA molecules attach to two regions: one near the N-terminus (LRR-NT and LRR1-LRR3) and one at the C-terminus (LRR19-LRR21). While protein–protein interactions only take place at LRR-CT in the TLR3-dsRNA complex, surface contacts (primarily hydrogen bonds and electrostatic interactions) are the only means by which TLR3 attaches to its receptors. To ensure TLR3 communication and dsRNA binding, the C-terminal dimerization region is essential [26].

The TRIF for downstream type I IFN signaling is shared by TLR3 and the DEAD (Asp-Glu-Ala-Asp) box polypeptide 1 (DDX1), DDX21, and DHX36 components of the DExD/H-box helicase cytosolic receptors of dsRNA. The only TLR receptor that relies exclusively on TRIF to trigger IFN-β release is TLR3. The TLR3-mediated signaling pathway can be divided into the TRIF-dependent nuclear transcription factor-κB (NF-κB) pathway and the TRIF-dependent IFN-regulatory factor 3/7 (IRF3/7) pathway based on the various downstream products that TRIF activates. For example, IRF3 induces the expression of type I interferons to mediate antiviral effects by activating other genes such as MxA genes [27].

TNF receptor-associated factor 3 (TRAF3) and TRAF6 interact with TRIF once it has been triggered in the plasmalemma by exogenous dsRNA to initiate a sequence of cascade events [28]. IRF3/7, NF-kB and activator protein 1 (AP-1) are the transcription factors that this signaling pathway eventually engages in, causing the production of type I IFNs (IFN-I), pro-inflammatory cytokines, and chemokines after TLR3 activation, respectively. TLR3 signaling also activates the proteins phosphoinositide 3-kinase (PI3K), p38-mitogen-activated protein kinase (MAPK), extracellular signal-regulated kinase (ERK), and c-Jun N-terminal kinase (JNK). Fine-tuning of ubiquitination and phosphorylation is essential for the TLR3 signaling cascade [29].

The meta-analysis carried out in this study explored the correlation between the rs3775291 polymorphism of the *TLR3* gene and the risk of infection and disease development in different countries. Analyzing the six studies carried out on the European continent, most of them (83.33%) have shown an association between the presence of the ancestral allele (C) and the risk of infection, while one of them detected that the mutant allele (T) may be involved with the risk of developing a disease, as seen in BKPyV infection after kidney transplantation [30]. Regarding the analysis of data from three American [31,32,33] and nine Asian studies [13,24,34,35,36,37,38,39,40], a significantly higher risk of infection with these diseases was found.

An essential line of defense in innate immunity related to the blood–brain barrier (BBB) is provided by a subpopulation of human neurons that expresses TLR3 on a constitutively. These hypotheses that TLR3 expression facilitates TBEV penetration through the BBB, which promotes the onset of neurologic illness, but also serves as a protective mechanism during established central nervous system (CNS) infection, may help explain these seemingly contradictory findings regarding the presence of this *TLR3* SNP [41]. Other investigations were conducted to examine the role of this SNP in responses to viral infections on the European continent [42,43]. These findings imply that in cases of TBE infection and HIV infection, the mutation may inhibit *TLR3* signaling activity, inhibiting viral entry into the central nervous system.

SNPs that cause loss of function of *TLR3*, such as rs3775291, appear to have an impact on the ability of the CNS to withstand HSV infection [44]. Studies in rodents show that astrocytes lacking TLR3 cannot produce an IFN-α response to HSV-2, predisposing these animals to an increased HSV-2 CNS infection if the peripheral (genital) infection does not worsen [45]. However, fibroblasts from TLR3-deficient patients with HSV-1 encephalitis (HSE) have abolished type I IFN activation, in contrast to PBMCs from the same patients, which respond properly to identical stimuli. Therefore, it is possible that the main function of TLR3 is to cause a type I IFN response to HSV-2 in non-hematopoietic cells rather than to support the acquired immune system [46].

According to a Spanish study, individuals who are homozygous carriers of the SNP T minor (TT) allele rs3775291 are two times more likely to develop BK polyomavirus (BKPyV) viremia [30]. TLR3 is involved in the stimulation of innate immune mechanisms during antiviral and inflammatory responses to BKPyV. Uncontrolled signaling caused by an SNP, such as rs3775291, can have an impact on the pathogenesis of BKPyV-associated nephropathy (BKPyVAN) by reducing its signaling activity compared to the wild-type type [25].

The study by Chen et al. (2017) makes additional assumptions that the missense polymorphism in rs3775291 may improve the anticancer immunostimulatory role (hepatocellular carcinoma—HCC linked to HBV) and support the apoptotic process [39].

The relationship between the *TLR3* rs3775291 polymorphism and HBV infection has been studied in Chinese populations [35,38]. Wan et al. (2016) were unable to find any evidence linking SNP to intrauterine HBV infection [38]. However, another study found that the mutant T allele was significantly more common in people with chronic hepatitis B and acute chronic renal failure, suggesting that this polymorphism may be a risk factor for the progression of the illness [35].

TLR3 has been associated with the release of cytokines and cellular activation caused by HCV [31]. On the other hand, the TLR3 ligand inhibits HBV proliferation in the liver of HBV mutant mice, according to recent investigations on infection [47]. The *TLR3* SNP rs3775291 in this situation impairs the secretion of the TLR3 ectodomain and reduces the receptor’s ability to respond to antigenic stimuli from these viruses [48].

Arboviral outbreaks have a significant negative impact on the population of Latin America [49]. There have been investigations into the potential effects of the rs3775291 polymorphism on the clinical forms of arboviruses. Santos et al. (2019) examined the relationship between SNP and microcephaly caused by the Zika virus. The results of the study demonstrated a link between the existence of the T mutant allele in the SNP rs3775291 in the *TLR3* gene and the risk of congenital Zika syndrome (CZS) in pregnant women who contracted the virus during pregnancy. This link can be explained by the decline in the function of phenylalanine-containing *TLR3* (T allele), and it directly interferes with antiviral activity, leading to an increase in viral load and making it easier for the pathogen to enter the developing brain [32].

The Asian continent produced the majority of the studies found examining the relationship between the *TLR3* SNP rs3775291 and viral susceptibility and the progression of patient cases. An analysis of how this polymorphism affected dengue cases in India was performed. The study by Alagarasu et al. (2015) that involved the Indian population found that the minor allele T has a negative impact on the presence of the protein structure and may be directly related to a reduction in inflammation, which would protect against the progression of dengue infections [36].

In addition to that, the measles virus (MV) appears to reach the CNS at the time of initial infection. Microglia and astrocytes, endogenous brain cells, are key players in the immunological responses that occur in this area. Type I IFN is induced by MV infection through TLR3, and in virus-infected cells, type I IFN is significantly upregulates TLR3 translation in a positive feedback way. Previous studies have shown that type I IFN inhibits efficient MV replication in rodents. Therefore, immunological dysregulation in measles is produced by uncontrolled signaling of TLR3 mediated by this polymorphism [24,50].

Furthermore, there are strong associations between this *TLR3* SNP and the differences in the downstream intracellular signaling molecules Myeloid Differentiation factor 2 (MD-2) and Myeloid Differentiation Primary Response 88 (MyD88) in both antibody and cellular responses to measles immunization [51]. The links between TLR3 and measles vaccination immunity are particularly intriguing because TLR3 had been found to be a main target for laboratory-adapted measles virus strains, but not for wild-type measles virus strains, in the generation of host immunity. Lab-adapted and vaccine-derived measles virus isolates, such as Edmonston, up-regulate TLR3 expression in human dendritic cells through enhanced IFN-β release according to Tanabe et al. (2003) [50].

Exon 1 is 500 bp upstream the region of the *TLR3* gene that reacts to the measles virus. The region contains the binding sites for the transcription factors NF-kB and STAT (a family of eukaryotic transcription factors that mediates the response to a variety of cytokines and growth factors) and the interferon-stimulated response element (ISRE), also known as an IFN-β induction site, is located 30 bp upstream of exon 1 in the promoter region of the *TLR3* gene [51].

Once the IFN response is activated, the transcript levels of more than 300 genes (ISGs) rise. These genes create proteins with immunoregulatory and antiviral properties that, in some cases, can stop the spread of viruses and reduce their growth. MxA, OAS, and A3G are recognized as reliable markers of IFN activity and are particularly important in the immunological intracellular response. One of the molecular mechanisms by which HTLV-1 counteracts and evades the IFN system is the cellular protein SOCS, which suppresses STAT1 phosphorylation and blocks intracellular signal transduction downstream of the IFN receptor, IFNAR1/2, in CD4+ cells from HTLV-1-infected individuals. When cells infected with HTLV-1 are cultured in vitro, Tyk2 and STAT2, two essential molecules in the initiation chain of the IFN pathway, have lower levels of phosphorylation. Additionally, when p30 is present, the interferon response is suppressed during viral replication, which contributes to the inhibition of TLR3 signaling [52].

These differential outcomes between infections and this SNP in different global populations can be traced to each individual’s particular immune response, which is closely related to his or her genetic background. Studies in immunogenetics are currently very helpful to determine the roles in the vulnerability of infection prevention, as they consider genetic background variables [53]. This highlights the need for more research to elucidate the precise function of SNP alleles in the susceptibility or protection of viral diseases.

Regarding SARS-CoV-2, Dhangadamajhi et al. (2021) examined the potential relationship between this *TLR3* variant and COVID-19 based on open international genomic databases of world populations and came to the conclusion that SNP is related to susceptibility to disease and mortality [54]. Although flaws in the analysis were discovered, Pati et al. (2021) revealed these facts in a publication so that the scientific community can carefully assess the findings of the previous article [55]. A five-year-old Brazilian male patient who was the subject of another case study was thought to be susceptible to hepatitis C due to SARS-CoV-2, but the genotyping of the SNP under discussion in this analysis failed to identify the mutant allele (T) in this person [56]. Due to the applied methodology, letters to the editor and case studies were excluded from this meta-analysis.

This present meta-analysis is a pioneering investigation of the evaluation of infections, in general. However, previous meta-analyses have already examined associations between this SNP and particular infections, including one that reported a relationship between this SNP and HIV-1 (which played a protective role for this infection) [57]; the significant association between the mutant allele and the risk of HBV infection [58]; an estimate of an almost two-fold increase in the risk effect for both HBV and HCV infections [59].

This study is constrained by the following factors: (1) the variation in definitions of disease cases from various studies; (2) the heterogeneity of the SNP acting as a potential bias in characteristics such as ethnicities and ages of different populations due to the phenomenon of a genetic background; (3) the methodology used; (4) the need to evaluate the data of interactions between gene and environmental factors.

## 5. Conclusions

The SNP of the *TLR3* gene, rs3775291, which is related to viral infections, has been linked to the protection, susceptibility, and severity of a number of diseases. Although this polymorphism has been extensively investigated, there is debate over which allele would be linked to the severe form of the illness, as in the case of studies on TBEV infection.

Despite this, the meta-analysis allows the verification of SNP behavior between populations, which can be linked to susceptibility in the cases of Americans and Asians or protection against disease in the case of those living on the European continent. To further understand the role of this SNP in infection and the development of novel pharmaceutical medicines that aid in the treatment. Further research focusing on various diseases, particularly those caused by viruses and the analysis of people from other countries, may be helpful.

## Figures and Tables

**Table 1:** Characteristics of studies included in this review for *TLR3* SNP rs3775291.

Reference/Database	Type of Infectious Agent	Methodology/Genotyping Method/Population Size	Country/Continent/Ethnic Group	Gender Ratio (Male/Female)/Average Age of Participants	Absolute Count of Alleles and Genotypes (Cases/Controls)	HWE	*p*-Value	Results
Ishizaki et al. (2008) [20]/Science Direct	Virus/ssRNA-	Case-control/TaqMan essays/124 subjects (40 patients and 84 controls)	Japan/Asia/Not reported.	Male: 27/Female: 13 and 84 children (with a no reported gender); Age: 12.4 years	T alleles (33/46) Dominant Model Genotypes (17/29)	*p* > 0.05	*p* = 0.03	The mutant T allele of the SNP has been associated with the risk of subacute sclerosing panencephalitis (SSPE) related to measles virus persistence.
Kindberg et al. (2011) [21]/PUBMED	Virus/ssRNA+	Case-control/PCR Genotyping/340 subjects (128 patients and 212 controls)	Lithuania/Europe/Not reported.	Gender ratio and average age not reported.	T alleles (59/141) Dominant Model Genotypes (50/101)	*p* > 0.05	*p* < 0.05	The wild-type C allele has been associated with the risk of contracting tick-borne encephalitis virus (TBEV).
Svensson et al. (2012) [22]/PUBMED	Virus/dsDNA	Case-control/TaqMan Assay/401 individuals (239 patients and 162 controls)	Sweden/Europe/Not reported.	Cases (Male: 136; Female: 103)/Controls (Male: 88; Female: 74); Age: 38 years.	T alleles (120/109) Dominant Model Genotypes (106/87)	*p* > 0.05	*p* = 0.0272	This SNP conferred protection against herpes simplex virus type 2 (HSV-2).
Lee; Brown; Razonable (2013) [23]/PUBMED	Virus/ssRNA+	Case-control/PCR Genotyping/611 subjects (153 patients and 458 controls)	USA/North America/Not reported.	Male: 395/Female: 216; Age: 52 years	T alleles (102/237) Dominant Model Genotypes (88/207)	*p* > 0.05	*p* = 0.03	The presence of the SNP promoted susceptibility to the Hepatitis C virus (HCV).
Li; Zheng (2013) [24]/PUBMED	Virus/dsDNA	Case-control/PCR Genotyping/948 subjects (466 patients and 482 controls)	China/Asia/Not reported.	Cases (Male: 383/Female: 83); Controls (Male: 386/Female: 96); Age: 53.8 years	T alleles (326/249) Dominant Model Genotypes (274/226)	*p* > 0.05	*p* = 0.004	It is a risk factor for developing HBV infection.
Rong et al. (2013) [25]/PUBMED	Virus/dsDNA	Case-control/PCR Genotyping/914 subjects (452 patients and 462 controls)	China/Asia/Not reported.	Cases (Male: 340/Female: 112)/Controls (Male: 344/Female: 118); Age: 38.88 years	T alleles (296/235) Model Genotypes (254/212)	*p* > 0.05	*p* = 0.002	The SNP increased the risk of HBV infection.
Huik et al. (2013) [26]/PUBMED	Virus/ssRNA+	Case-control/TaqMan Assay/842 subjects (172 HIV-positive patients and 670 controls)	Estonia/Europe/White.	Cases (Male: 133/Female: 69)/Controls (Male:Not reported/Female: Not reported); Age: 30 years.	T alleles (108/455) Dominant Model Genotypes (92/376)	*p* > 0.05	*p* = 0.03	The SNP promoted HIV-1 protection.
Mickienė et al. (2014) [27]/PUBMED	Virus/ssRNA+	Case-control/PCR Genotyping/560 subjects (348 patients and 212 controls)	Lithuania/Europe/Not reported.	Cases (Male: 195/Female: 154); Controls (Male: Not reported/Female: Not reported). Age—Children TBE (Cases: 12.07/Controls: 11.43 years) Age—Adult severe TBE (Cases: 51.93 ± 15.419/Controls: 57.27 ± 15.108) Age—Adult TBE (Cases: 43.56/Controls: 46.97 years)	T alleles (195/141) Dominant Model Genotypes (157/101)	*p* > 0.05	*p* = 0.02	The SNP confers less risk of getting TBEV infection.
Alagarasu et al. (2015) [28]/PUBMED	Virus/ssRNA+	Case-control/PCR-RFLP/229 subjects (120 patients and 109 controls)	India/Asia/Not reported.	Cases (Male:73/Female: 47)/Controls (Male: 67/Female: 42); Age: 31.3 years	T alleles (59/63) Dominant Model Genotypes (52/48)	*p* > 0.05	*p* = 0.04	The SNP confers a greater risk of acquiring the dengue virus.
BiyaniByyani et al. (2015) [29]/PUBMED and Science Direct	Virus/ssRNA+	Case-control/PCR Genotyping/206 individuals (103 patients and 103 controls)	India/Asia/Not informed.	Gender ratio not reported; Age: 18.04.	T alleles (60/33) Dominant Model Genotypes (45/28)	*p* > 0.05	*p* = 0.013	The SNP has been associated with the risk of Japanese encephalitis virus (JEV) infection.
Wan et al. (2016) [30]/PUBMED	Virus/dsDNA	Case-control/TaqMan Assay/563 subjects (35 patients and 528 controls)	China/Asia/Not reported.	Cases (Male: 18/Female: 26)/Controls (Male: 358/Female: 295); Age: 26.43 years.	T alleles (17/329) Dominant Model Genotypes (17/282)	*p* > 0.05	*p* = 0.736	No significant association for SNP and HBV infection.
Chen et al. (2017) [31]/PUBMED	Virus/dsDNA	Case-control/PCR Genotyping/978 subjects (292 patients and 686 controls)	China/Asia/Not reported.	Male: 686/Female: 0; Age: 37 years.	T alleles (240/235) Dominant Model Genotypes (195/415)	*p* > 0.05	*p* = 0.0001	The SNP is a protective factor for HBV infection.
Grygorczuk et al. (2017) [32]/PUBMED	Virus/ssRNA+	Case-control/TaqMan Assay/180 subjects (108 patients and 72 controls)	Poland/Europe/Not informed.	Gender: Not reported; Age: 42.44 years.	T alleles (59/41) Dominant Model Genotypes (51/36)	*p* > 0.05	*p* < 0.05	The higher frequency of wild-type C allele in patients was associated with TBEV infection.
Santos et al. (2019) [33]/PUBMED	Virus/ssRNA+	Case-control/Quantitative Real Time-PCR/255 subjects (168 patients and 87 controls)	Brazil/South America/Not reported.	Not reported.	T alleles (94/43) Dominant Model Genotypes (83/37)	*p* > 0.05	*p* = 0.042	The SNP increased risk of developing Zika virus infection.
Posadas-Mondragón et al. (2020) [34]/PUBMED	Virus/ssRNA+	Case-control/Real Time PCR/211 participants (165 patients and 46 controls)	Mexico/North America/Not reported.	DEN: 88/77; GP: Not reported; Age—Cases: 39.85, Controls: 49.52.	T alleles (96/56) Dominant Model Genotypes (83/23)	*p* > 0.05	*p* = 0.31	The mutant T allele of this SNP has been associated with protection from the dengue virus.
Habibabadi et al. (2020) [13]/PUBMED	Virus/ssRNA+	Case-control/PCR-RFLP/218 subjects (100 patients and 118 controls)	Iran/Asia/Not reported.	Male: 179/Female: 39; Age—Case Group: 38.55, Control Group: 36.72 years.	T alleles (53/70) Dominant Model Genotypes (48/63)	*p* > 0.05	*p* = 0.46	No association between this SNP and human T-cell lymphotropic virus type 1 (HTLV-1).
Singh et al. (2021) [35]/PUBMED	Virus/ssRNA+	Case-control/PCR Genotyping/196 individuals (98 patients and 98 controls)	India/Asia/Not informed.	Cases (Male: 36/Female: 62)/Controls (Male: 39; Female: 59); Age: 37.7 years	T alleles (52/28) Dominant Model Genotypes (42/25)	*p* > 0.05	*p* = 0.10	The presence of the mutant T allele of this SNP was associated with dengue susceptibility.
Redondo et al. (2022) [36]/PUBMED	Virus/dsDNA	Case-control/TaqMan Assay/204 individuals (50 cases and 154 controls)	Spain/Europe/Not reported.	Male: 146/Female: 58; Age: 54.6 years	T alleles (39/90) Dominant Model Genotypes (28/75)	*p* > 0.05	*p* = 0.029	Homozygous carriers of the T minor allele (TT genotype) of the highlighted SNP had a twofold increased risk of BK polyomavirus viremia (BKPyV) after kidney transplantation.

## Data Availability

The original contributions of the study are included in the article. Further inquiries can be directed to the corresponding authors.
